# Unlocking Youth Creativity: The Power of Socioemotional Skills

**DOI:** 10.3390/children13020261

**Published:** 2026-02-13

**Authors:** Cátia Branquinho, Catarina Noronha, Marina Carvalho, Nuno Neto Rodrigues, Margarida Gaspar de Matos

**Affiliations:** 1 Aventura Social, University of Lisbon, 1400-415 Lisbon, Portugal; cbranquinho@ucp.pt (C.B.); catarinanoronha26@live.com.pt (C.N.); marina.carvalho@ismat.pt (M.C.); 2 Faculdade de Ciências Humanas, University Católica Portuguesa, Palma de Cima, 1649-023 Lisbon, Portugal; 3 Institute of Environmental Health (ISAMB), University of Lisbon, 1649-004 Lisbon, Portugal; 4 Department of Psychology and Physical Education, Instituto Superior Manuel Teixeira Gomes (ISMAT), 8500-590 Portimão, Portugal; 5 Direção Geral de Estatísticas da Educação e Ciência, Avenida 24 de Julho 134, 1200-774 Lisbon, Portugal; nuno.rodrigues@dgeec.medu.pt; 6 Applied Psychology Research Centre Capabilities and Inclusion, Instituto Superior de Psicologia Aplicada, 1140-041 Lisbon, Portugal

**Keywords:** creativity, socioemotional competences, school-aged children, positive youth development, well-being, age and gender differences

## Abstract

Background/Objectives: Creativity has become an essential skill for children and adolescents to cope with the challenges of contemporary society. Beyond academic success, creativity is closely linked to well-being, social adjustment, and personal development. Schools, therefore, play a crucial role in creating conditions that allow students to explore ideas, express themselves, and develop socioemotional resources. This study aimed to examine how self-perceived creativity relates to educational, socioemotional, and well-being factors in Portuguese students, to identify different creativity profiles, and to explore the main variables that predict creativity. Methods: This cross-sectional study was based on secondary analyses of national data from the project Psychological Health and Well-being|School Observatory. The sample included 3011 students aged between 9 and 20 years (M = 13.62; SD = 2.53), from grades 5 to 12. Data were collected using validated instruments: the OECD Socioemotional Skills Survey (SSES), the Positive Youth Development (PYD) scale, and the WHO-5 Well-Being Index. Analyses included group comparisons, cluster analysis to identify self-perceived creativity profiles, correlation analyses, and multiple regression models. Results: Self-perceived creativity did not differ between boys and girls, but it decreased significantly with higher grade levels. Three profiles were identified: low, medium, and high self-perceived creativity. Students with higher self-perceived creativity reported better well-being, more positive relationships with teachers, a stronger sense of belonging at school, and higher parental educational levels. Self-perceived creativity was positively associated with socioemotional skills such as curiosity, sociability, and optimism, as well as with PYD dimensions and well-being. Negative associations were found with age and test anxiety. Socioemotional variables were the strongest predictors of creativity, explaining 39% of its variance. Conclusions: These results show that creativity is closely connected to students’ socioemotional development. Investing in emotional skills, supportive relationships, and positive school environments may be a powerful way to foster creativity and promote healthier, more balanced development. This has important implications for educational practice and policy.

## 1. Introduction

### 1.1. The Relevance of Creativity in the 21st Century

In the 21st century, there has been widespread recognition of the importance of creativity as a crucial talent for the growth, well-being, and survival of school-aged children. Education plays a vital role in fostering and enhancing creative abilities, especially as the world faces numerous complex challenges that require innovative solutions [1,2].

In educational research, creativity is increasingly understood not only as the production of original and useful outcomes, but also as a developmental and dispositional process grounded in an individual’s personally meaningful interpretations of experience and in their self-perceptions of creative potential [3]. In the present study, creativity is conceptualized as self-perceived creativity, assessed through the SSES|Creativity subscale, capturing students’ subjective evaluation of their creative tendencies rather than objective creative performance. This approach aligns with a domain-general and socioemotional perspective of creativity, embedded in everyday school functioning rather than domain-specific artistic or academic production.

According to Kupers et al. [2], creativity can be understood as a human characteristic that develops over time through exposure to new ideas and processes, involving collaborative relationships among peers, teachers, and the surrounding environment. The iterative creative process uses feedback from one iteration to inform the next. This perspective supports the view of creativity as a dynamic, socially embedded competence that can be nurtured through educational experiences and socioemotional development.

### 1.2. Creativity in Educational Contexts

To nurture and support student creativity, a supportive learning environment is essential. In a systematic review, van der Zanden et al. [4] identified four categories of factors related to creativity: individual, parental, educational, and social. Richardson and Mishra [5] assert that learning engagement, the teaching environment, and physical surroundings can all influence students’ creative thinking. Every subject has the potential to foster creativity, and as Albar and Southcott [6] demonstrate, the aim of education should be to impart knowledge while also fostering critical thinking skills. In schools, students who receive only direct instruction may become less creative and more passive learners [2]. The pro-creativity pedagogical method, according to Cremin and Chappell [7], comprises seven interconnected features: idea generation and exploration, playfulness, problem-solving, risk-taking, co-construction and collaboration, and teacher creativity. Together, these features emphasize that creativity is not merely an individual attribute, but a pedagogical and relational process shaped by classroom climate, teacher practices, and opportunities for student agency and exploration.

Furthermore, Barbot et al. [8] identify three reasons for differences in originality among young people: task specificity, personal resources, and environmental effects. This framework highlights that creativity emerges from the interaction between individual characteristics (such as cognitive abilities, personality traits, and motivation), the nature of the tasks students engage in, and the quality of the surrounding educational and social context.

According to the literature, schools serve as the main setting for delivering universal early interventions to school-aged children, as nearly all children attend school [9]. Over the past decade, there has been a growing adoption of universal, school-based socioemotional learning programs, aimed at strengthening children’s social and emotional skills to support their mental health [10,11,12]. This reinforces the relevance of studying creativity within a socioemotional framework, as creativity is increasingly viewed as intertwined with emotional regulation, motivation, engagement, and well-being.

### 1.3. Individual and Contextual Factors Associated with Creativity

Regarding age, research identifies developmental “slumps and pumps” contingent upon specific contextual and cultural needs. It has been proposed that as people age and acquire new skills, their creativity may increase [8]. A study by Razumnikova et al. [13] found that young people outperformed children in verbal and figurative creativity as well as fluid intelligence, although age-related variations in creativity depend on the situation. Furthermore, a longitudinal study by Claxton et al. [14] examining the cognitive and affective aspects of creativity in children from the fourth to the ninth grades found that divergent thinking and divergent emotion increase throughout life.

These findings suggest that developmental changes in creativity are not linear and may be reflected both in chronological age and in school grade, which represents a key educational and social context for development. In the present study, grade level is used to capture educational-stage differences, whereas age is considered a continuous developmental indicator. Additionally, studies indicate that individuals with higher creativity levels are better equipped to handle perceived stress in challenging situations. This ability to manage stress effectively is linked to experiencing more positive emotions, leading to the conclusion that creativity is associated with enhanced personal well-being [15].

Overall, socioemotional skills tend to develop exponentially during the transition from childhood to adolescence [16]. These skills will accompany young people throughout their development and are important to the consolidation of their personality and behaviors [16]. The development of socioemotional skills appears in the literature as a promoter of good development and better adaptation of children and young people to the demands inherent to development [17]. This highlights the potential role of socioemotional competencies as central mechanisms through which creativity is expressed and supported.

### 1.4. Interconnections Between Creativity, Socioemotional Competencies, Well-Being, and Positive Youth Development

The literature suggests that enhancing socioemotional skills can promote creative thinking and self-directed learning, which are essential components of positive youth development [18]. Fostering socioemotional skills—creativity, in particular—contributes to resilience and the ability to cope with challenges, thereby promoting their well-being and supporting positive youth development [19]. From this perspective, creativity can be understood both as an outcome of socioemotional development and as a resource that strengthens adaptive functioning, autonomy, and engagement.

The novelty of the present study lies in the integration of self-perceived creativity with a comprehensive socioemotional framework (SSES), subjective well-being (WHO-5), and Positive Youth Development (PYD), using a large national dataset. In addition, the study adopts a profiling approach that identifies distinct creativity profiles and examines developmental trends across school grades, which remains underexplored in previous research using similar frameworks.

This study aims to examine self-perceived creativity in children and adolescents, investigating its variation by demographic factors such as gender, grade level, and parental education. Additionally, it explores how socioemotional competencies (SSES), well-being (WHO-5) [20], and Positive Youth Development (PYD) resources relate to and predict SSES|Creativity. The analyses follow a sequential approach, starting with descriptive and inferential assessments, then identifying creativity profiles through cluster analysis, and finally conducting regression analyses to determine key predictors of SSES|Creativity.

Based on previous literature, we hypothesize that:

(i)   Self-perceived creativity will decline across school grades, reflecting developmental “slumps”;
(ii)  No substantial gender differences will be observed;
(iii) Socioemotional competencies will emerge as stronger predictors of creativity than demographic variables, well-being, or PYD indicators.

## 2. Materials and Methods

### 2.1. Participants

This study included 3011 school-aged children, 1522 girls (50.5%) and 1489 boys (49.5%), aged 9 to 20 years old (*M* = 13.62; *SD* = 2.532), from grades 5 to 12 in schools across mainland Portugal. Regarding school level, 25.6% were enrolled in lower secondary education (5th and 6th grades; ages ± 11–12), 40.7% in middle secondary education (7th to 9th grade; ages ± 13–15), and 33.7% in upper secondary education (10th to 12th grade; ages ± 16–18). The vast majority (92.5%) held Portuguese nationality.

With respect to the educational level of the father, 39.1% had completed upper secondary education, 34.2% had completed basic education, 21.6% held a higher education degree, and 5.1% had not completed any level of formal education. Regarding the mother, 38.8% had completed upper secondary education, 34.3% had completed higher education, 23.6% had completed basic education, and 3.4% had not completed any level of formal education. Although schools were selected through a stratified sampling procedure (see Section 2.2), the final sample should be interpreted as not fully representative due to non-proportional stratification, voluntary participation, and missing data. Sample size varied across analyses due to item-level missingness; the effective N is reported for each analysis.

Although the sampling framework was designed to ensure geographical coverage through stratified school selection, the final analytical sample cannot be considered fully representative of the Portuguese student population due to the non-proportional nature of the stratification, voluntary participation at both school and student levels, the absence of sampling weights, and missing data; therefore, representativeness applies to the school selection strategy but not necessarily to the final sample composition. Information regarding the number of schools and classes invited versus those that effectively participated was not systematically recorded, which prevents the calculation of precise response rates. This constitutes an additional limitation of the study.

### 2.2. Procedure

Under the oversight of the Portuguese Ministry of Education, the research project titled “Psychological Health and Well-being|School Observatory” was created in collaboration with the following organizations: the National Program for the Promotion of School Success, the Portuguese Psychologists Association, the Calouste Gulbenkian Foundation, the Aventura Social Team, and the Directorate-General for Education and Science Statistics.

The sample consisted of a stratified (non-proportional) random selection of public mainland schools, stratified by geographic region/NUTS III (Nomenclature of Territorial Units for Statistics). Data were collected between 23 January and 9 June 2024. Following the stratified random selection of classes by grade level, the teachers and school psychologists responsible for the participating schools administered the data collection instruments in computer labs. Students completed the questionnaires exclusively online, with prior informed consent obtained from their parents or legal guardians. The administration protocol lasted on average between 20 and 30 min. The methods and results are described in detail in the online study report, available at https://info.dgeec.medu.pt/saude-psicologica-e-bem-estar-2024/ (accessed on 10 December 2025) or Matos et al. [21].

Regarding ethics and data protection, data collection was conducted under the authorization and oversight of the Directorate-General for Education and Science Statistics (DGEEC), within the Portuguese Ministry of Education. Participation was voluntary and required informed consent from parents/legal guardians, and student assent when appropriate. Data were collected anonymously online and processed in accordance with the GDPR and the Declaration of Helsinki.

### 2.3. Measures

The primary goal of the “Psychological Health and Well-being|School Observatory” was to examine the key variables associated with psychological health and well-being in three student age groups: middle school students (self-reports), high school students (self-reports), and preschool and elementary school students (reports by teachers/educators). In addition to student data, self-report information was also collected from teachers, school management staff, educational psychologists, support technicians, operational assistants, and parents or legal guardians, offering a comprehensive perspective on the school ecosystem.

Only data from lower secondary education to upper secondary education were used in the present study. The research protocol included individual variables related to psychological health and well-being, along with sociodemographic questions. Several standardized instruments were employed (Table 1 and Table 2).

One of the main tools used was the Socioemotional Skills Survey (SSES), developed by the Organisation for Economic Co-operation and Development [22], from which a wide range of variables were drawn. These included the following subscales: SSES|Optimism, Emotional regulation, Resilience, Trust, Confidence, Curiosity, Sociability, Persistence, Creativity, Energy, Cooperation, Self-control, Sense of belonging to school, Bullying, Relationship with teachers, and Test anxiety. For all subscales, higher values indicate higher levels of the respective competence, except for Bullying, where higher scores reflect greater victimization experiences, and Test Anxiety, where higher scores reflect higher levels of anxiety.

The SSES Creativity subscale assesses students’ self-perceived tendency to generate new ideas, approach problems in original ways, and engage in imaginative thinking in everyday contexts. All SSES subscales were computed as mean scores across items (range 0–4). Negatively worded items were reverse-coded prior to score computation, following the official OECD scoring guidelines for the SSES instrument.

In addition, the WHO-5 Well-Being Index [20] was applied to assess students’ subjective well-being. Finally, the Positive Youth Development (PYD) [23] measure was used, based on the Portuguese-validated version of the instrument [24], ensuring cultural and contextual adequacy. Additionally, the variables of gender, school grade, father’s educational level, and mother’s educational level were also used.

### 2.4. Statistical Procedures

The data were analyzed using SPSS version 29.0 (SPSS Inc., Chicago, IL, USA). Statistical analysis was conducted in several stages. Initially, the SSES|Creativity subscale was examined descriptively by gender and grade level. Analysis of Variance (ANOVA) revealed significant differences between grade levels, and Tukey’s HSD post hoc tests were applied to identify which specific grades differed.

Additionally, we tested the Gender × Grade interaction; as it was not statistically significant, results are presented as main effects.

Next, a non-hierarchical cluster analysis (K-means) was performed using standardized SSES|Creativity scores to group participants into homogeneous profiles. A three-cluster solution was selected to facilitate the interpretation and comparison of low, moderate, and high creativity profiles. Subsequently, clusters were compared on SSES variables, WHO-5, PYD dimensions, and parental education using ANOVAs.

Because clustering was based on a single variable, this procedure is conceptually similar to empirically derived cut-points (e.g., tertiles). This approach was used to support profile comparisons, and its limitations are acknowledged. The clustering approach was preferred over arbitrary cut-off methods (such as tertiles or ±1 SD) because it allows for empirically derived group boundaries based on the actual distribution of creativity scores in the sample.

To characterize and compare the creativity profiles, a one-way ANOVA was applied. Tukey’s HSD post hoc tests were used to determine which cluster pairs showed statistically significant differences. When the homogeneity of variances was violated (Levene’s test *p* < 0.05), the Games–Howell post hoc test was used because it is robust to unequal variances and unequal group sizes.

Finally, to identify the main predictors of SSES|Creativity, a hierarchical multiple regression was conducted. The model was built in three stages: (i) demographic variables (grade level, gender, and parental education) were introduced; (ii) all SSES were added; (iii) Well-being|WHO-5 and PYD were incorporated.

Spearman’s rho correlations were computed because parental education is an ordinal variable, and a preliminary inspection indicated deviations from normality in several distributions. Normality was assessed through the inspection of skewness and kurtosis values and visual examination of histograms.

Missing data were handled using listwise deletion within each analysis; therefore, sample sizes varied across ANOVA, cluster, correlation, and regression models depending on data availability. The effective sample size was *N* = 3011 for descriptive analyses, *N* = 2734 for cluster analyses, and *N* = 2517 for regression models.

All analyses were conducted with a 95% confidence level, ensuring the robustness and reliability of the results.

## 3. Results

### 3.1. Gender Differences in SSES|Creativity

ANOVA showed no statistically significant differences between genders on this subscale. Initially, the SSES|Creativity subscale was analyzed according to the participants’ gender. The obtained means indicated similar levels of self-perceived creativity between boys (*M* = 2.54; *SD* = 0.64) and girls (*M* = 2.51; *SD* = 0.67).

### 3.2. SSES|Creativity Across Grade Levels

Subsequently, the variable grade level, ranging from 5th to 12th grades, was examined. Results showed that students in 5th grade had the highest mean score on the SSES|Creativity (*M* = 2.76; *SD* = 0.72), whereas the 9th grade group registered the lowest mean (*M* = 2.4; *SD* = 0.60). ANOVA revealed statistically significant differences between school grades (*F*(7, 2921) = 10.41, *p* < 0.001, *η*^2^ = 0.024), suggesting a significant association between school grade and SSES|Creativity. Although statistically significant, this effect size is small, indicating that grade level explains a limited proportion of the variance in self-perceived creativity.

To identify where these differences occurred, Tukey’s HSD post hoc tests were conducted. The results showed that students in the 5th grade scored significantly higher than those in the 8th grade (Mdiff = 0.31, *p* < 0.05), 9th grade (Mdiff = 0.36, *p* < 0.001), 10th grade (Mdiff = 0.26, *p* < 0.05), 11th grade (Mdiff = 0.30, *p* < 0.05), and 12th grade (Mdiff = 0.27, *p* < 0.05). No significant differences were found between the 5th and 6th grades.

Similarly, students in the 6th grade showed significantly higher creativity scores than those in the 8th grade (Mdiff = 0.14, *p* < 0.05), 9th grade (Mdiff = 0.19, *p* < 0.01), 11th grade (Mdiff = 0.13, *p* < 0.05), and 12th grade (Mdiff = 0.12, *p* < 0.05).

Taken together, these findings suggest that self-perceived creativity tends to be higher in the early school years, particularly in the 5th and 6th grades, and gradually decreases in later grades, with the lowest values observed in the 9th grade. The remaining grades (7th, 8th, 10th, 11th, and 12th) showed intermediate mean scores, falling between the higher levels of the 5th grade and the lower levels of the 9th grade, which is consistent with the descriptive results presented in Table 3 and Table 4.

### 3.3. Identification of SSES|Creativity Profiles: Cluster Analysis

To identify homogeneous groups of participants, a non-hierarchical cluster analysis (K-means) was conducted based on the SSES|Creativity subscale. The final clusters, using standardized scores (Z-scores) from the SSES|Creativity subscale, were as follows:

- Cluster 1 (Low Creativity): *Z* = −1.456
- Cluster 2 (High Creativity): *Z* = 1.168
- Cluster 3 (Moderate Creativity): *Z* = −0.290

These clusters represent distinct creativity profiles: Cluster 1 includes participants with scores significantly below the sample mean (*n* = 1064), Cluster 2 includes those with scores significantly above the mean (*n* = 461), and Cluster 3 comprises individuals whose scores are close to the overall sample average (*n* = 1209).

Before examining differences between creativity profiles across socioemotional, well-being, and developmental variables, a one-way ANOVA was conducted to confirm that the identified clusters differed significantly in creativity. Results revealed a significant effect of cluster membership on creativity, *F*(2, 2731) = 5706.73, *p* < 0.001, with a very large effect size (*η*^2^ = 0.81). This represents an extremely large effect size, confirming the strong differentiation between the three creativity profiles. Post hoc comparisons using the Games–Howell test indicated that all three clusters differed significantly from each other (*p* < 0.001), supporting the distinctiveness of the low, moderate, and high creativity profiles.

The next step involved conducting Analyses of Variance (ANOVAs) to compare the mean scores of the clusters across various SSES. The ANOVA results revealed significant differences between the clusters for all variables assessed (*p* < 0.05). Tukey HSD post hoc tests showed the following patterns:

- Cluster 2 (High Creativity) consistently showed the highest mean scores across almost all SSES indicators, Well-being|WHO-5, and PYD variables, except for SSES|Bullying and SSES|Test anxiety, with significant differences from the other clusters (*p* < 0.001).
- Cluster 1 (Low Creativity) had the lowest mean scores on most SSES indicators, Well-being|WHO-5, and PYD, with significant differences from Clusters 2 and 3 (*p* < 0.05), except for a few variables.
- Cluster 3 (Moderate Creativity) fell between the other clusters, with mean scores significantly higher than Cluster 1 and lower than Cluster 2 for most variables (*p* < 0.05).

Notable exceptions were observed in a few specific variables. In terms of SSES|Resilience/stress resistance, no significant difference was found between Clusters 1 and 3; however, both differed significantly from Cluster 2, which exhibited higher resilience levels. Regarding SSES|Bullying, Cluster 2 demonstrated significantly lower levels of victimization compared to Clusters 1 and 3, between which no significant differences emerged. For SSES|Test anxiety, participants in Cluster 1 reported significantly higher anxiety than those in Cluster 2, while no significant differences were found between Clusters 1 and 3. Finally, in relation to Parents’ educational level, only Cluster 2 stood out, presenting significantly higher values than Clusters 1 and 3, which did not differ from one another (Table 5).

**Figure 1:**
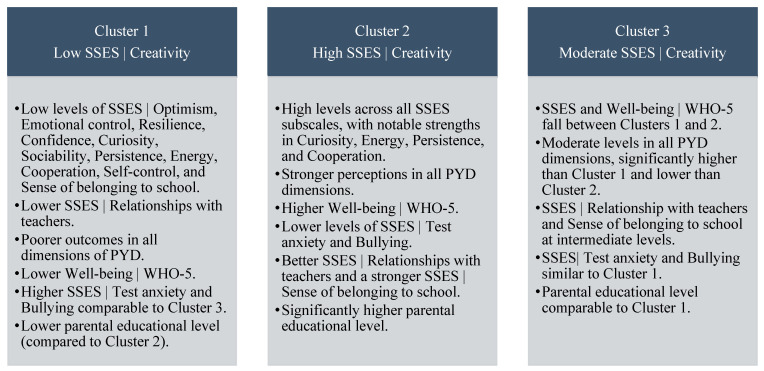
Conceptual summary of the low, moderate, and high creativity profiles.

Based on these results, the competencies associated with each creativity profile can be summarized as follows (Figure 1):

### 3.4. Correlations with SSES|Creativity

School grade was used as the primary developmental indicator in group comparisons because it reflects students’ educational context, whereas age was included in correlational analyses as a continuous developmental variable.

Correlational analysis was subsequently conducted to examine the direction and magnitude of the associations between SSES|Creativity and the other study variables, serving as the statistical basis for the hierarchical regression that followed.

This analysis revealed a significant negative correlation between SSES|Creativity and age (*ρ* = −0.107, *p* < 0.001), indicating that younger students tend to report higher levels of perceived creativity.

Regarding parental education level, significant positive correlations were observed with SSES|Creativity for both the father’s level of education (*ρ* = 0.134, *p* < 0.001) and that of the mother (*ρ* = 0.129, *p* < 0.001). This suggests that a higher level of parental education is associated with higher levels of creativity in students.

The SSES subscales showed strong relationships with SSES|Creativity, with the most notable being SSES|Curiosity (*ρ* = 0.507, *p* < 0.001), SSES|Energy (*ρ* = 0.458, *p* < 0.001), SSES|Cooperation (*ρ* = 0.459, *p* < 0.001), SSES|Persistence (*ρ* = 0.447, *p* < 0.001), SSES|Optimism (*ρ* = 0.368, *p* < 0.001), SSES|Sociability (*ρ* = 0.394, *p* < 0.001), SSES|Emotional regulation (*ρ* = 0.251, *p* < 0.001), and SSES|Resilience (*ρ* = 0.153, *p* < 0.001).

Additionally, SSES|Creativity was positively correlated with SSES|Sense of belonging to school (*ρ* = 0.319, *p* < 0.001), SSES|Relationship with teachers (*ρ* = 0.192, *p* < 0.001), SSES|Self-control (*ρ* = 0.384, *p* < 0.001), and well-being measured by the Well-being|WHO-5 (*ρ* = 0.234, *p* < 0.001). Conversely, a negative correlation was found between SSES|Creativity and SSES|Bullying (*ρ* = −0.091, *p* < 0.001), while a non-significant negative correlation was observed with SSES|Test anxiety.

Within the PYD framework, SSES|Creativity showed significant positive associations with the dimensions of PYD|Competence (*ρ* = 0.373, *p* < 0.001), PYD|Confidence (*ρ* = 0.387, *p* < 0.001), PYD|Connection (*ρ* = 0.366, *p* < 0.001), and PYD|Contribution (*ρ* = 0.177, *p* < 0.001).

### 3.5. Predictors of SSES|Creativity: Hierarchical Multiple Regression

A hierarchical multiple regression was conducted to assess the predictive capacity of demographic variables, SSES, PYD, and Well-being|WHO-5 on the dependent variable SSES|Creativity.

In Model 1, demographic variables (grade level, gender, and parental education) were entered as control variables. This model was statistically significant (*F*(4, 2512) = 20.897, *p* < 0.001), explained only 3.2% of the total variance in SSES|Creativity (*R*^2^ < 0.05). Within this block, both grade level and parental education significantly predicted SSES|Creativity. Grade level showed a negative association with SSES|Creativity (*β* = −0.092, *p* < 0.001), whereas parental education was positively associated (father’s education: *β* = 0.07, *p* < 0.01; mother’s education: *β* = 0.091, *p* < 0.001). Gender did not emerge as a significant predictor of SSES|Creativity.

In Model 2, the SSES subscales were added, resulting in a substantial and statistically significant increase in explained variance (*ΔR*^2^ = 0.359, *p* < 0.001). The full model accounted for 39.1% of the variance in SSES|Creativity (*F*(18, 2498) = 89.168, *p* < 0.001). Examination of standardized coefficients revealed several SSES as robust predictors of SSES|Creativity, even after controlling for demographic variables. The strongest positive predictors were SSES|Curiosity (*β* = 0.26, *p* < 0.001), SSES|Energy (*β* = 0.193, *p* < 0.001), SSES|Cooperation (*β* = 0.178, *p* < 0.001), and SSES|Sociability (*β* = 0.11, *p* < 0.001). Conversely, SSES|Trust (*β* = −0.100, *p* < 0.001) and SSES|Test anxiety (*β* = −0.052, *p* < 0.01) were significant negative predictors. The inclusion of these socioemotional variables diminished the predictive power of most demographic variables, with grade level and father’s education level ceasing to be significant predictors.

Finally, Model 3 incorporated Well-being|WHO-5 and PYD variables. However, this addition did not lead to a significant increase in explained variance, indicating that Well-being|WHO-5 and PYD factors did not uniquely contribute to predicting SSES|Creativity beyond the socioemotional competencies already accounted for. Only mother’s education level remained positively and significantly associated with SSES| Creativity (*β* = 0.04, *p* < 0.05) (Table 6 and Table 7).

## 4. Discussion

The results of this study reveal that SSES|Creativity does not show significant variations between genders, in line with the results of Ogurlu et al. [25] and Nakano et al. [26]. The latter concluded that there is no consensus on gender differences, suggesting that aspects such as opportunities, motivation, and attitudes should be considered a priority. This finding reinforces the idea that creativity-related self-perceptions are not inherently gendered, but rather shaped by contextual and educational opportunities.

With regard to grade level, there is a statistically significant but small decline in SSES|Creativity as students progress through their academic career, especially between 5th and 9th grades. Although statistically significant, the magnitude of this effect is modest (η^2^ = 0.024), suggesting that the observed decline reflects subtle developmental changes rather than a marked loss of creative capacity. This pattern may reflect a global trend of declining skills such as curiosity, creativity, and openness to experience in early adolescence [27,28]. This result is consistent with the literature that identifies creative “slumps” at this stage [7], attributed to cognitive, emotional, and social changes typical of adolescence, although some studies point to creative “pumps” associated with the acquisition of new skills and life experiences. In the present study, this potential increase may be related to the age of entry into secondary school.

This decline highlights a critical window for intervention, underscoring the importance of pedagogical strategies that preserve and stimulate creativity from the age of 11–12. Rather than indicating a disappearance of creativity, this pattern may reflect changes in school demands, evaluation practices, and motivational climate that progressively constrain students’ opportunities for creative expression. Although the literature points to the positive effects of implementing creativity promotion programs in schools [29,30], their effective integration continues to face significant barriers, such as curriculum overload, the absence of creativity assessment indicators, and insufficient teacher training and capacity building [31]. Models such as the “7Cs of Creativity” [32] and the “4C Model” [3] offer solid theoretical frameworks to guide these practices, emphasizing the multidimensional and developmental nature of creativity and the need for intentionally structured educational contexts to enhance it. In this sense, the study by Li et al. [33] highlights the emerging role of digital technologies as promising resources to support creative development, especially in interactive learning environments, paving the way for educational practices more aligned with the challenges of the 21st century.

The regression analysis also revealed that dimensions such as SSES|Curiosity, Energy, and Cooperation are the most robust predictors of SSES|Creativity, confirming the relational and developmental nature of this competency. SSES|Creativity should not be seen as a fixed trait, but rather as a dynamic capacity, influenced by other SSES subscales and susceptible to being enhanced through deliberate pedagogical practices [34]. From a developmental and social-cognitive perspective, curiosity reflects intrinsic motivation and openness to experience, which are central mechanisms for creative exploration and idea generation. Energy may be interpreted as an indicator of engagement and vitality that sustains creative effort, whereas cooperation highlights the fundamentally social nature of creativity in school contexts, where ideas are often co-constructed through interaction. These findings are consistent with cognitive approaches to creativity that highlight the contribution of higher-order cognitive processes, including mental imagery and the manipulation of mental representations, to creative expression. Empirical studies have shown that such cognitive resources are associated with creative performance, suggesting that socioemotional competencies such as curiosity and engagement may support the cognitive mechanisms involved in everyday creativity [35,36,37]. In fact, studies show a significant association between curiosity and creativity [38], reinforcing the relevance of educational contexts that value exploration, questioning, and reflection. The role of energy/vitality as a creative facilitator [39,40] and cooperation as a catalyst for collective creativity [32,41] reinforces the need for learning strategies that integrate intrinsic motivation, collaborative work, and real-world challenges.

In addition, the data show that SSES|Creativity is positively associated with Well-being|WHO-5 and other socioemotional skills, such as SSES|Emotional regulation, Resilience, and Sense of belonging to school. Students with higher levels of SSES| Creativity perceive themselves as more resilient, emotionally balanced, and integrated into the school context. This result reinforces the literature that associates creativity with positive emotions, reduced perceived stress [15], and greater adaptability [42,43]. The link found between SSES|Creativity and PYD indicators supports the idea that promoting creative skills contributes not only to academic performance but also to the construction of positive identities, greater autonomy, and better social adaptation [19]. Carvalho et al. [44] also showed that adolescents with higher levels of PYD|Competence, Confidence, and Connection reported fewer psychological symptoms and greater life satisfaction, highlighting how PYD dimensions are closely related to well-being outcomes. The literature also shows that SSES|Creativity and Resilience share traits such as flexibility, problem-solving skills, and adaptability [45,46]. However, the absence of a unique contribution of WHO-5 and PYD in the final regression model suggests that their relationship with creativity is largely explained by shared variance with socioemotional skills, indicating that SSES subscales capture core psychological processes linking creativity, well-being, and positive development. The role of the family also emerges as a determining factor in students’ creative profile, with parental involvement being fundamental in stimulating curiosity, autonomy, and emotional expression.

In short, the data from this study point to the need for a systemic and integrated approach to promoting creativity, linking schools, families, and communities. Promoting SSES|Creativity involves investing in the cross-curricular development of socioemotional skills and student well-being, creating consistent and equitable learning opportunities throughout the school career. Integrating creativity into curricula, in line with the “4Cs” of 21st-century learning (Critical Thinking, Communication, Collaboration, and Creativity), can not only enhance academic performance [47] but also prepare young people to face the complex challenges of the present and the future in an innovative, ethical, and resilient way [48].

### 4.1. Strengths and Limitations

The study benefits from a large sample size, which enhances the reliability and generalizability of the results, although the sample is not representative of the population. Its methodological approach is comprehensive and robust, combining cluster analyses and hierarchical multiple regression. This combination allowed for not only the identification of SSES|Creativity profiles but also their validation and the effective determination of their predictors. Furthermore, the variety of variables examined—SSES, Well-being (WHO-5), and PYD—provides a rich and multifaceted context for interpreting the results.

The main limitation lies in the cross-sectional design, which prevents causal inference. The observed decline in SSES|Creativity across advancing school years, for example, is a comparative observation rather than a true longitudinal development. Additionally, SSES|Creativity was assessed through self-report, which may not correspond to objective creativity. Finally, it is important to note that although some associations (such as with education level) were statistically significant, their effect sizes were modest, indicating a limited influence on the variance in creativity. Moreover, students were nested within classes and schools, which may violate the assumption of independence of observations. As multilevel modeling was not applied, potential clustering effects could not be statistically controlled. The use of a one-variable cluster solution also limits the conceptual richness of the profiles, which should be interpreted as empirical groupings rather than multidimensional typologies. Moreover, the large number of statistical tests increases the risk of Type I error, and the substantial reduction in sample size across analyses due to missing data may have affected statistical power and generalizability.

### 4.2. Implications for the Future and Public Policy

- Based on the present findings, school-based programs targeting socioemotional skills appear to be promising avenues for supporting the development of SSES|Creativity from the early years of elementary education, particularly before the 7th grade. These programs may benefit from curricular approaches that prioritize methodologies fostering curiosity, cooperation, and active engagement.
- Educational policies should support teacher training initiatives that strengthen key socioemotional skills related to creativity, such as curiosity, energy, cooperation, emotional regulation, resilience, persistence, and sense of belonging to school, while fostering safe school environments that value emotional well-being, psychological safety, and positive relationships.
- Family involvement should be encouraged, particularly in contexts of lower parental educational levels, to strengthen children’s cultural and social capital. In addition, monitoring systems for SSES|Creativity and related competencies may help tailor interventions and support students’ holistic development.

## 5. Conclusions

This study highlights creativity as a socioemotional and developmental competence that is strongly embedded in students’ everyday school experiences. Rather than being primarily shaped by demographic characteristics, self-perceived creativity appears to be closely linked to socioemotional skills such as curiosity, energy, and cooperation, reinforcing the idea that creativity emerges in relational and motivational contexts.

Although a small decline in creativity was observed across grade levels, particularly between the 5th and 9th grades, this finding should be interpreted as a subtle developmental shift rather than a loss of creative potential. It points to early adolescence as a sensitive period in which educational environments may play a crucial role in sustaining students’ creative engagement. Overall, the results support an understanding of creativity as a dynamic capacity that can be nurtured through educational practices that promote socioemotional development, well-being, and positive relationships within the school community. Future research using longitudinal and multilevel designs will be essential to deepen the understanding of how creativity evolves over time and across educational contexts.

### Key Messages

(1) Self-perceived creativity shows a small but significant decline between 5th and 9th grades, identifying early adolescence as a sensitive period for educational support.
(2) Socioemotional skills, particularly curiosity, energy, and cooperation, are the strongest predictors of creativity.
(3) Creativity is closely linked to students’ well-being and positive development, reinforcing its relevance for holistic education.

## Figures and Tables

**Table 1:** Description of the instruments and variables assessed in the study, including the number of items and response formats.

Variable	Number of Items	Scale
Gender	1	1 = Male and 2 = Female
School grade	1	1 = 5th grade; 2 = 6th grade; 3 = 7th grade; 4 = 8th grade; 5 = 9th grade; 6 = 10th grade; 7 = 11th grade; 8 = 12th grade
Parental educational level	1	1 = no completed school level; 2 = basic education; 3 = upper secondary education; 4 = higher education
SSES|Socioemotional Skills		
Optimism	8	0 = Strongly disagree to 4 = Strongly agree
Emotional regulation	8	
Resilience	8	
Trust	8	
Curiosity	8	
Sociability	8	
Persistence	8	
Creativity	8	
Energy	8	
Cooperation	8	
Self-control	8	
Sense of belonging to school	6	
Bullying	4	0 = Never or almost never to 3 = Once a week or more
Relationship with teachers	3	
Test anxiety	3	0 = Strongly disagree to 4 = Strongly agree
PYD|Positive Youth Development		
Competence	6	(used for most items): 0 = Strongly disagree to 4 = Strongly agree (used only for items i and j): 0 = Never true to 4 = Always true
Confidence	6	
Connection	8	
Contribution	5	0 = No time to 4 = 6 or more hours
Well-being|WHO-5	5	0 = Never to 5 = All of the time

Note: SSES—Socioemotional Competences Scale; PYD—Positive Youth Development.

**Table 2:** Measurement levels, ranges, descriptive statistics, and reliability of the study variables.

Variable	Level of Measurement	Range	M	SD	Cronbach’s Alpha
Gender	Nominal	1–2	1.51	0.5	--
School grade	Ordinal	1–8	4.39	2.23	--
Age	Interval	9–20	13.62	2.53	--
Father’s educational level	Ordinal	1–4	2.77	0.84	--
Mother’s educational level	Ordinal	1–4	3.04	0.85	--
SSES|Creativity (dependent variable)	Interval	0–4	2.52	0.66	0.83
SSES|Optimism	Interval	0–4	2.71	0.77	0.85
SSES|Emotional regulation	Interval	0–4	2.15	0.73	0.77
SSES|Resilience	Interval	0–4	2.05	0.86	0.88
SSES|Trust	Interval	0–4	2.12	0.73	0.85
SSES|Curiosity	Interval	0–4	2.63	0.69	0.85
SSES|Sociability	Interval	0–4	2.49	0.72	0.82
SSES|Persistence	Interval	0–4	2.59	0.7	0.85
SSES|Energy	Interval	0–4	2.37	0.7	0.81
SSES|Cooperation	Interval	0–4	2.91	0.64	0.88
SSES|Self-control	Interval	0–4	2.5	0.65	0.84
SSES|Sense of belonging to school	Interval	0–4	2.47	0.53	0.76
SSES|Bullying	Interval	0–3	0.44	0.68	0.84
SSES|Relationship with teachers	Interval	0–3	2.32	0.78	0.81
SSES|Test anxiety	Interval	0–4	2.49	1.05	0.85
PYD|Competence	Interval	0–24	14.38	4.7	0.84
PYD|Confidence	Interval	0–24	15.17	5.44	0.9
PYD|Connection	Interval	0–30	18.86	5.7	0.85
PYD|Contribution	Interval	0–20	8.87	4.4	0.72
Well-being|WHO-5	Interval	0–25	15.73	4	0.84

**Table 3:** Descriptive statistics for the creativity subscale by grade level.

Grade Level	*N*	*Mean*	*SD*
5th	330	**2.76**	**0.72**
6th	410	2.59	0.70
7th	411	2.55	0.64
8th	374	2.45	0.65
9th	415	2.40	0.60
10th	307	2.50	0.65
11th	371	2.46	0.58
12th	311	2.49	0.64

Note: Bold—highest score.

**Table 4:** Tukey post hoc test results for the Creativity subscale between grade levels.

Comparison	Mean Difference
5th vs. 8th	0.31 *
5th vs. 9th	0.36 ***
5th vs. 10th	0.26 *
5th vs. 11th	0.3 *
5th vs. 12th	0.27 *
6th vs. 8th	0.14 *
6th vs. 9th	0.19 **
6th vs. 11th	0.13 *
6th vs. 12th	0.12 *

Note: * *p* < 0.05, ** *p* < 0.01, *** *p* < 0.001.

**Table 5:** Descriptive statistics and ANOVA results by cluster.

Variable	Cluster 1 (*M*, *SD*)	Cluster 2 (*M*, *SD*)	Cluster 3 (*M*, *SD*)	*F*	η^2^	Post Hoc (Tukey HSD)
SSES|Optimism	2.25 (0.83)	**3.03 (0.71)**	2.66 (0.7)	183.27 ***	0.112	C2 > C3 > C1
SSES|Emotional regulation	1.88 (0.74)	**2.36 (0.81)**	2.11 (0.64)	70.2 ***	0.046	C2 > C3 > C1
SSES|Resilience	1.89 (0.92)	**2.23 (0.91)**	1.99 (0.78)	32.71 ***	0.022	C2 > (C1 = C3)
SSES|Trust	1.83 (0.72)	**2.29 (0.8)**	2.11 (0.65)	62.31 ***	0.041	C2 > C3 > C1
SSES|Curiosity	2.13 (0.77)	**3.03 (0.62)**	2.54 (0.57)	353.07 ***	0.195	C2 > C3 > C1
SSES|Sociability	2.05 (0.75)	**2.79 (0.74)**	2.43 (0.61)	185.6 ***	0.113	C2 > C3 > C1
SSES|Persistence	2.18 (0.72)	**2.95 (0.69)**	2.50 (0.59)	245.96 ***	0.144	C2 > C3 > C1
SSES|Creativity	1.91 (0.65)	**2.76 (0.74)**	2.27 (0.56)	294.97 ***	0.169	C2 > C3 > C1
SSES|Energy	2.57 (0.78)	**3.25 (0.55)**	2.81 (0.56)	242.55 ***	0.143	C2 > C3 > C1
SSES|Cooperation	2.18 (0.70)	**2.78 (0.67)**	2.43 (0.55)	166.3 ***	0.103	C2 > C3 > C1
SSES|Self-control	2.26 (0.52)	**2.66 (0.57)**	2.42 (0.47)	105.64 ***	0.068	C2 > C3 > C1
SSES|Bullying	0.48 (0.65)	0.37 (0.59)	0.48 (0.71)	8.65 ***	0.006	C2 < (C1 = C3)
SSES|Relationship with teachers	2.14 (0.88)	**2.49 (0.7)**	2.28 (0.77)	36.63 ***	0.025	C2 > C3 > C1
SSES|Test anxiety	**2.61 (1.13)**	2.41 (1.14)	2.50 (0.97)	5.36 **	0.004	C1 > C2, (C1 = C3), (C2 = C3)
PYD|Competence	11.61 (5.09)	**16.26 (4.5)**	14.05 (4.22)	168.74 ***	0.104	C2 > C3 > C1
PYD|Confidence	11.87 (5.72)	**17.46 (5.29)**	14.74 (4.84)	185.59 ***	0.113	C2 > C3 > C1
PYD|Connection	15.63 (6.16)	**21.07 (5.66)**	18.51 (4.96)	157.95 ***	0.098	C2 > C3 > C1
PYD|Contribution	7.64 (4.46)	**9.67 (4.22)**	8.75 (4.39)	32.9 ***	0.022	C2 > C3 > C1
Well-being|WHO-5	13.83 (5.25)	**17.06 (4.75)**	15.45 (4.86)	66.9 ***	0.045	C2 > C3 > C1
Father’s educational level	2.67 (0.81)	**2.94 (0.84)**	2.7 (0.83)	26.64 ***	0.019	C2 > (C1 = C3)
Mother’s educational level	2.97 (0.86)	**3.22 (0.81)**	2.95 (0.85)	30.67 ***	0.021	C2 > (C1 = C3)

Note: Bold—higher mean; ** *p* < 0.01, *** *p* < 0.001.

**Table 6:** Summary of hierarchical regression models predicting Creativity.

Model	R	R^2^	Adjusted R^2^	ΔR^2^	F Change	df1	df2
1	0.179	0.032	0.031 ***	0.032	20.897	4	2512
2	0.625	0.391	0.387 ***	0.359	105.207	14	2498
3	0.627	0.393	0.387	0.002	1.487	5	2493

Note: *N* = 2517. Model 1 includes grade level, gender, and parental education level. Model 2 adds SSES subscales. Model 3 adds Well-being|WHO-5 and all PYD dimensions. *** *p* < 0.001.

**Table 7:** Standardized coefficients of hierarchical regression predicting Creativity.

Variable	*B*	*SE*	*β*	*t*
Model 1				
(Constant)	2.315 ***	0.072		31.93
Grade level	−0.027 ***	0.006	−0.092	−4.643
Gender	−0.018	0.026	−0.014	−0.711
Father’s educational level	0.054 **	0.019	0.07	2.868
Mother’s education level	0.07 ***	0.019	0.091	3.736
Model 2				
(Constant)	0.557 ***	0.099		5.624
Grade level	−0.005	0.005	−0.019	−1.076
Gender	−0.01	0.024	−0.007	−0.409
Father’s educational level	0.02	0.015	0.025	1.294
Mother’s educational level	0.031 *	0.015	0.041	2.091
SSES|Optimism	0.019	0.02	0.022	0.942
SSES|Emotional regulation	−0.019	0.021	−0.022	−0.94
SSES|Resilience	0.015	0.018	0.02	0.844
SSES|Trust	−0.091 ***	0.018	−0.1	−5.041
SSES|Curiosity	0.249 ***	0.02	0.26	12.28
SSES|Sociability	0.1 ***	0.022	0.11	4.64
SSES|Persistence	0.084 ***	0.02	0.089	4.162
SSES|Energy	0.18 ***	0.021	0.193	8.442
SSES|Cooperation	0.183 ***	0.025	0.178	7.447
SSES|Self-control	0.056 *	0.023	0.055	2.454
SSES|Sense of belonging to school	−0.039	0.028	−0.031	−1.381
SSES|Bullying	−0.006	0.018	−0.005	−0.314
SSES|Relationship with teachers	−0.013	0.015	−0.016	−0.909
SSES|Test anxiety	−0.032 **	0.012	−0.052	−2.735
Model 3				
(Constant)	0.609 ***	0.102		5.995
Mother’s education level	0.031 *	0.015	0.04	2.069
SSES|Trust	−0.092 ***	0.019	−0.101	−4.888
SSES|Curiosity	0.249 ***	0.02	0.260	12.242
SSES|Sociability	0.093 ***	0.022	0.102	4.133
SSES|Persistence	0.08 ***	0.020	0.086	3.987
SSES|Energy	0.173 ***	0.023	0.186	7.633
SSES|Cooperation	0.174 ***	0.025	0.169	6.977
SSES|Self-control	0.053 *	0.023	0.052	2.304
SSES|Test anxiety	−0.032 **	0.012	−0.052	−2.704
Well-being|WHO-5	−0.004	0.003	−0.033	−1.566
PYD|Competence	0.004	0.004	0.027	1.004
PYD|Confidence	0.004	0.003	0.033	1.216
PYD|Connection	0.001	0.003	0.005	0.195
PYD|Contribution	0.002	0.003	0.012	0.691

Note: Significant variables with *p* < 0.05 are presented in Models 2 and 3. The non-significant variables from Model 3 (except for those added in this step) were omitted for clarity. * *p* < 0.05; ** *p* < 0.01; *** *p* < 0.001.

## Data Availability

The datasets generated and/or analyzed during the current study are not publicly available but are available from the corresponding author upon reasonable request.
